# The miR-124-p63 feedback loop modulates colorectal cancer growth

**DOI:** 10.18632/oncotarget.16248

**Published:** 2017-03-16

**Authors:** Kuijie Liu, Hongliang Yao, Sanlin Lei, Li Xiong, Haizhi Qi, Ke Qian, Jiqiang Liu, Peng Wang, Hua Zhao

**Affiliations:** ^1^ Department of General Surgery, The Second Xiangya Hospital, Central South University, Changsha 410011, China

**Keywords:** miR-124, p63, feedback loop, cell growth, colorectal cancer

## Abstract

Among the diverse co-regulatory relationships between transcription factors (TFs) and microRNAs (miRNAs), feedback loops have received the most extensive research attention. The co-regulation of TFs and miRNAs plays an important role in colorectal cancer (CRC) growth. Here, we show that miR-124 can regulate two isoforms of p63, TAp63 and ΔNp63, via iASPP, while p63 modulates signal transducers and activators of transcription 1 (STAT1) expression by targeting miR-155. Moreover, STAT1 acts as a regulator of CRC growth by targeting miR-124. Taken together, these results reveal a feedback loop between miRNAs and TFs. This feedback loop comprises miR-124, iASPP, STAT1, miR-155, TAp63 and ΔNp63, which are essential for CRC growth. Moreover, this feedback loop is perturbed in human colon carcinomas, which suggests that the manipulation of this microRNA-TF feedback loop has therapeutic potential for CRC.

## INTRODUCTION

Transcription factors (TFs) are proteins that bind to specific nucleic acid sequences to regulate target gene expression at the transcriptional level [1]. microRNAs (miRNAs) play important roles in cell development and differentiation and have also been implicated in oncogenesis [2]. In recent years, the potential of miRNAs to regulate the gene expression has emerged as a tool for diagnosis of a number of diseased conditions, such as cardiovascular diseases, cancer, neurodegenerative disorders and infectious diseases [3]. The coordinated activity of miRNAs and TFs describes the mechanism of gene expression control. Moreover, to drive or repress the expression of particular miRNAs or TFs, these two elements coordinate to form autoregulatory feedback loops [4]. Such a loop is the one in which the expression of one particular component is directly affected by the presence or absence of another [5].

miR-124 has received a large amount of attention, and researchers have focused on its interactions with various TFs and its prominent role in CRC development. miR-124 suppresses the growth of human CRC via a PTB1/PKM1/PKM2 feedback cascade [6] or via the targeting of STAT3 [7]. In addition, accumulating evidence has revealed that miR-124 is regulated by several TFs, including HNFα4 and NF-κB [8, 9]. In a previous study, we showed that miR-124 regulates the growth of CRC cells via the direct targeting of iASPP [10]. It has also been revealed that iASPP may affect cellular senescence and cell cycle withdrawal through its ability to bind p63 and inhibit its transcriptional activity [11–13]. Hence, we speculate that miR-124 may influence p63 expression.

P63, a crucial TF plays a major role in the process of many cancers, has been reported to be associated with various miRNAs [14, 15]. In the context of squamous cell carcinoma, specific miRNAs, including miR-181a, miR-519a, miR-374a and miR-630, were identified as downstream targets of p-ΔNp63α [16]. Computational genomic analysis has been used to determine the number of miRNAs that are regulated by p63. HEK293 cells were transfected with two isoforms of p63 (TAp63 and ΔNp63), and increased levels of endogenous iASPP expression were observed at both the protein and mRNA levels. Moreover, the depletion of p63 in keratinocytes significantly reduced endogenous iASPP protein and mRNA expression but did not affect p53 expression [12], which suggests that iASPP and p63 are linked in an auto-regulatory feedback loop. Because p63 negatively regulates iASPP and plays a significant role in various cancers through its interactions with miRNAs [12], we speculated about the potential association between p63 and miR-124 and that these molecules may form a feedback loop that affects CRC development. However, this hypothesis required further investigation.

As reported by previous studies, p63 can bind to the promoter of miR-155 [17, 18]. MiR-155, which is a significant regulator in CRC development [14, 19, 20], has also been reported to be involved in the progression of several cancer types. Given these data, we assume that the p63/miR-155 axis together with miR-124 might form a feedback loop by binding to other TFs. Using TargetScan and JASPAR, we found miR-155 binding sites in the 3′UTR of STAT1; we also found STAT1 binding sites in the promoter of miR-124. STAT1 has been regarded as a tumor suppressive TF [21, 22]. In the present study, we show that miR-124 regulates p63 via iASPP, while p63 targets miR-155 via the modulation of STAT1 expression. Moreover, STAT1 acts as a TF that binds to the promoter of miR-124, which leads to increases in p63 levels during feedback regulation. Taken together, these results support the presence of a miR-124-p63 feedback loop comprising iASPP, STAT1 and miR-155, which is essential for CRC development.

## RESULTS

### The miR-124/iASPP axis modulates cell growth in CRC via the regulation of p63

**Figure 1:**
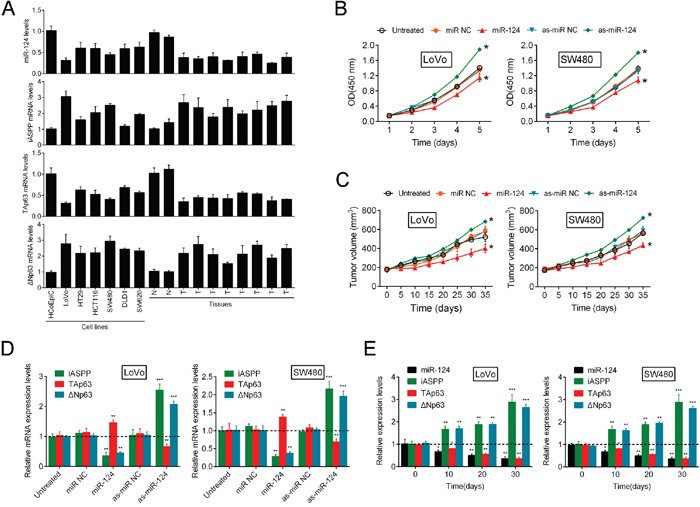
The miR-124/iASPP axis modulates CRC cell growth via the regulation of p63 **(A)** In the non-transformed immortalized human colon cell line HCoEpiC, six CRC cell lines, LoVo, HT29, HCT116, SW480, DLD1 and SW620, normal tissue samples and tumor tissue samples miR-124, iASPP, ΔNp63 and TAp63 expression was determined using real-time PCR. **(B, C)** The change of CRC cell growth (mean ± SD) and tumor formation in immunodeficient mice (mean ± SEM) was determined in response to inhibition of miR-124 and miR-124 overexpression. **(D)** The expression levels of iASPP, ΔNp63 and TAp63 were determined using real-time PCR in response to miR-124 overexpression and inhibition of miR-124. **(E)** The expression levels (mean ± SD) of iASPP, miR-124, TAp63 and ΔNp63 were examined in a time-dependent manner. The data are presented as the mean ± SD of three independent experiments. *P<0.05, **P<0.01, ***P<0.005.

To elucidate the role and function of the miR-124/iASPP axis and p63 in CRC growth, we determined the expression of miR-124, iASPP and two isoforms of p63 (TAp63 and ΔNp63) in the following samples: the non-transformed immortalized human colon cell line HCoEpiC; six CRC cell lines (LoVo, HT29, SW480, SW620, DLD1 and HCT116); and human tumor tissues (N stands for normal tissues and T stands for tumor tissues). We observed a decrease in miR-124 and TAp63 expression and an increase in iASPP and ΔNp63 expression in all six CRC cell lines and human tumors compared with the control (Figure 1A). Moreover, the expression differences of miR-124, iASPP, TAp63 and ΔNp63 were the most significant in LoVo and SW480 cell lines, compared with the normal cell line, HcoEpic. So the two cell lines were selected as the research objects for further experiments.

In our previous study, we identified the miR-124/iASPP axis as a regulator of CRC growth that involves the direct targeting of iASPP by miR-124. To confirm whether the miR-124/iASPP axis exerts its effect through p63, further studies were performed. MiR-124 inhibition/overexpression was achieved using as-miR-124/miR-124; the expression efficiency was verified using real-time PCR 48 h after transfection (Supplementary Figure 1). On day 5 and day 35 after tumor formation in nude mice, the expression levels of miR-124 were determined by RT-PCR (Supplementary Figure 2). Strikingly, stable cell line injection-induced inhibition of miR-124 was sufficient to elevate CRC cell growth and promote tumor formation in immunodeficient mice, while miR-124 overexpression strongly inhibited CRC cell proliferation and tumor formation (Figure 1B and 1C). In these tumors on day 35, miR-124 expression remained suppressed (Supplementary Figure 2).

Because the regulatory effect of miR-124 on CRC cell proliferation was confirmed, we further examined the expression levels of iASPP and p63 in response to miR-124 overexpression and inhibition. Recently, we described the regulation of CRC cell proliferation via the targeting of iASPP by miR-124 [10]. Consistent with our previous study, the present data indicated that miR-124 overexpression markedly decreased iASPP and ΔNp63 expression and enhanced TAp63 expression; in contrast, the inhibition of miR-124 resulted in a dramatic upregulation of iASPP and ΔNp63 expression and an attenuation of TAp63 expression (Figure 1D). To determine the expression correlation of iASPP, miR-124, TAp63 and ΔNp63 in tumor tissues derived from mice, we determined the expression levels of iASPP, miR-124, TAp63 and ΔNp63 over time. Significant downregulation of miR-124 and TAp63 expression was observed over time and was accompanied by the upregulation of iASPP and ΔNp63 expression (Figure 1E). This result is consistent with previous results obtained in six CRC cell lines and tissues and suggests that miR-124 regulates CRC cell proliferation through iASPP and p63. MiR-124 significantly promoted TAp63 protein expression while inhibited ΔNp63 protein expression. The overexpression of iASPP partially restored the regulatory effect of miR-124 on TAp63 and ΔNp63 protein (Supplementary Figure 3). Overall, these data suggest that miR-124 inhibition induces growth of CRC cells, and p63 and iASPP are involved in this process.

### Knockdown of p63 promotes CRC cell growth through miR-124/iASPP feedback

**Figure 2:**
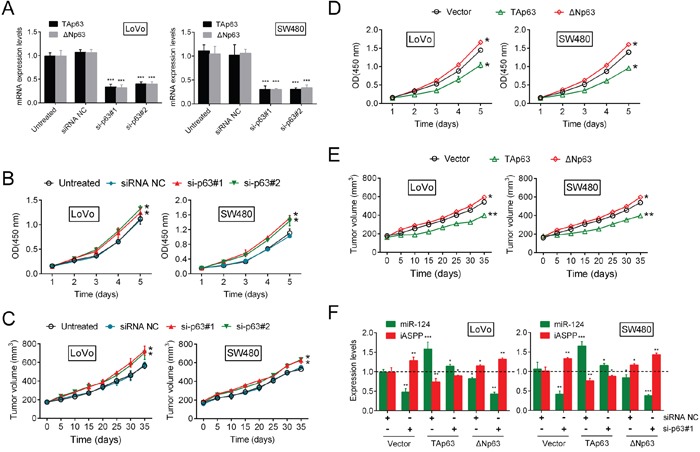
Knockdown of p63 promotes CRC cell growth through miR-124/iASPP feedback **(A)** Efficient p63 inhibition was verified. **(B)** The cell growth of both LoVo and SW480 cells (mean ± SD) was determined using the CCK-8 assay. **(C)** Tumor formation (mean ± SEM) was determined in response to p63 inhibition. **(D, E)** The CRC cell growth (mean ± SD) and the tumor volume (mean ± SEM) were determined in response to TAp63 or ΔNp63 treatment. **(F)** We co-treated the LoVo and SW480 cells with Vector + siRNA NC/si-p63#1, TAp63 + siRNA NC/si-p63#1 or ΔNp63 + siRNA NC/si-p63#1, and then the expression levels of miR-124 and iASPP (mean ± SD) were determined. The data are presented as the mean ± SD of three independent experiments. *P<0.05, **P<0.01, ***P<0.005.

Building on the *in vitro* and *in vivo* findings that the miR-124/iASPP axis regulates CRC cell proliferation via the inhibition of cell proliferation by TAp63 and the promotion of cell proliferation by ΔNp63, we hypothesized that p63 knockdown would result in negligible changes in cell proliferation; however, p63 knockdown significantly facilitated cell proliferation. This observation prompted us to assess the functional roles of TAp63 and ΔNp63 in the regulation of cell proliferation. The silencing of p63 was achieved through the use of si-p63#1 and si-p63#2, and efficient inhibition of TAp63 and ΔNp63 was then verified (Figure 2A, Supplementary Figure 4A). On day 5 and day 35 after tumor formation in nude mice, the expression levels of p63 were verified by RT-PCR (Supplementary Figure 5). The results of the CCK-8 assay showed that the growth of both LoVo and SW480 cells were induced in response to p63 inhibition (Figure 2B). Consequently, tumor formation was also promoted via suppression of the p63 levels (Figure 2C), which suggests that, between the two isoforms, TAp63 might play the leading role in this process. Based on the silencing of p63, we achieved TAp63 and ΔNp63 overexpression (Supplementary Figure 6A, 6B). The proliferation of CRC cells and the tumor volume were determined. The results showed that TAp63 treatment significantly suppressed p63 knockdown-induced CRC cell proliferation. In contrast, the upregulation of ΔNp63 amplified the promotive function of si-p63#1 on the proliferation of CRC cells and tumor formation (Figure 2D, 2E). Moreover, we transfected the LoVo and SW480 cells with Vector (empty vector used for control), TAp63 or ΔNp63 in the presence or absence of si-p63#1, and then the expression levels of miR-124 and iASPP were determined using real-time PCR. TAp63 induction rescued the si-p63#1 effect on the expression of miR-124 and iASPP. ΔNp63 overexpression also decreased miR-124 levels and increased the iASPP levels, but did not rescue the effect of si-p63#1 (Figure 2F). Taken together, the data discussed above suggested that TAp63 plays a leading role in the regulation of CRC growth through p63 via the mediation of the negative feedback regulation of miR-124/iASPP expression. However, it has been reported that ΔNp63 can promote the binding of p63 to the miR-155 promoter, although it has not been demonstrated to regulate miR-155 expression [18].

### p63 regulates miR-155 expression through the targeting of the miR-155 host gene

**Figure 3:**
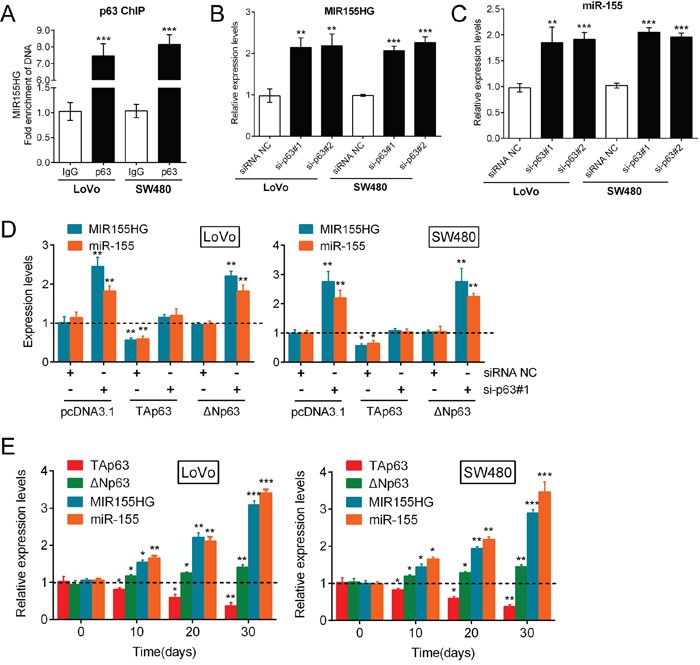
p63 regulates miR-155 expression by targeting the miR-155 host gene **(A)** ChIP was used to subsequently demonstrate that endogenous p63 was directly recruited to this consensus p63-RE in LoVo and SW480 cells. **(B, C)** The expression levels of both the MIR155HG transcript and mature miR-155 were determined in response to knockdown of p63 by si-p63#1 and si-p63#2. **(D)** TAp63 overexpression markedly restored miR-155 expression, while ΔNp63 overexpression resulted in minimal changes in miR-155 expression. The data are presented as the mean ± SD of three independent experiments. **(E)** The expression levels (mean ± SD) of TAp63, ΔNp63, MIR155HG transcript and mature miR-155 in mice tumors were examined every 10 days for 30 days. The data are presented as the mean ± SD of three independent experiments. *P<0.05, **P<0.01, ***P<0.005.

As a vital transcription factor, p63 is involved in the regulation of various miRNAs including miR-155. Mattiske et al. reported that the expression levels of miR-155 were significantly decreased by the overexpression of TAp63, while the overexpression of ΔNp63 did not significantly change the expression levels of miR-155 [18]. In addition, we revealed the dominant role of TAp63 in the regulation of CRC cell growth. Hence, we speculated that p63 regulates miR-155 expression during CRC growth. First, we examined the roles of TAp63 and ΔNp63 in the transcriptional control of MIR155 Host Gene (MIR155HG). An *in silico* analysis using p63 Scan software (Radboud University, The Netherlands) identified a putative p63 response element (RE) in the third exon of the MIR155HG gene. ChIP was performed, which demonstrated that endogenous p63 was directly recruited to the predicted consensus p63-RE of MIR155HG gene in LoVo and SW480 cells (Figure 3A). The knockdown of p63 by either si-p63#1 or si-p63#2 significantly increased the expression levels of both the MIR155HG transcript and the mature miR-155 (Figure 3B, 3C). Then, we examined the levels of both the MIR155HG transcript and mature miR-155 in response to TAp63 and ΔNp63 knockdown by si-p63#1, and overexpression in CRC cells. The results showed that MIR155HG transcript and mature miR-155 could be significantly promoted by si-p63#1 transfection, down-regulated by TAp63, whereas not affected by ΔNp63; after p63 knockdown, forced TAp63 expression markedly reversed the effect of si-p63#1 on miR-155 and MIR155HG expression; after p63 knockdown, ΔNp63 overexpression could not reverse the effect of si-p63#1 on miR-155 and MIR155HG expression (Figure 3D). These suggested that the two isoforms of p63 might exert different functions in regulating miR-155 expression: TAp63 suppressed miR-155 expression while ΔNp63 promoted miR-155 expression. In addition, we examined the expression of TAp63, ΔNp63, MIR155HG transcript and mature miR-155 in mouse tumors every 10 days for 30 days. The results showed that TAp63 expression was reduced, while the expression levels of ΔNp63, MIR155HG transcript and mature miR-155 were all significantly increased (Figure 3E). Therefore, p63 regulates miR-155 expression through the targeting of MIR155HG, and the TAp63 isoform plays a dominant role in this process.

### miR-155 targets STAT1 and consequently modulates the miR-124/iASPP/p63 pathway

**Figure 4:**
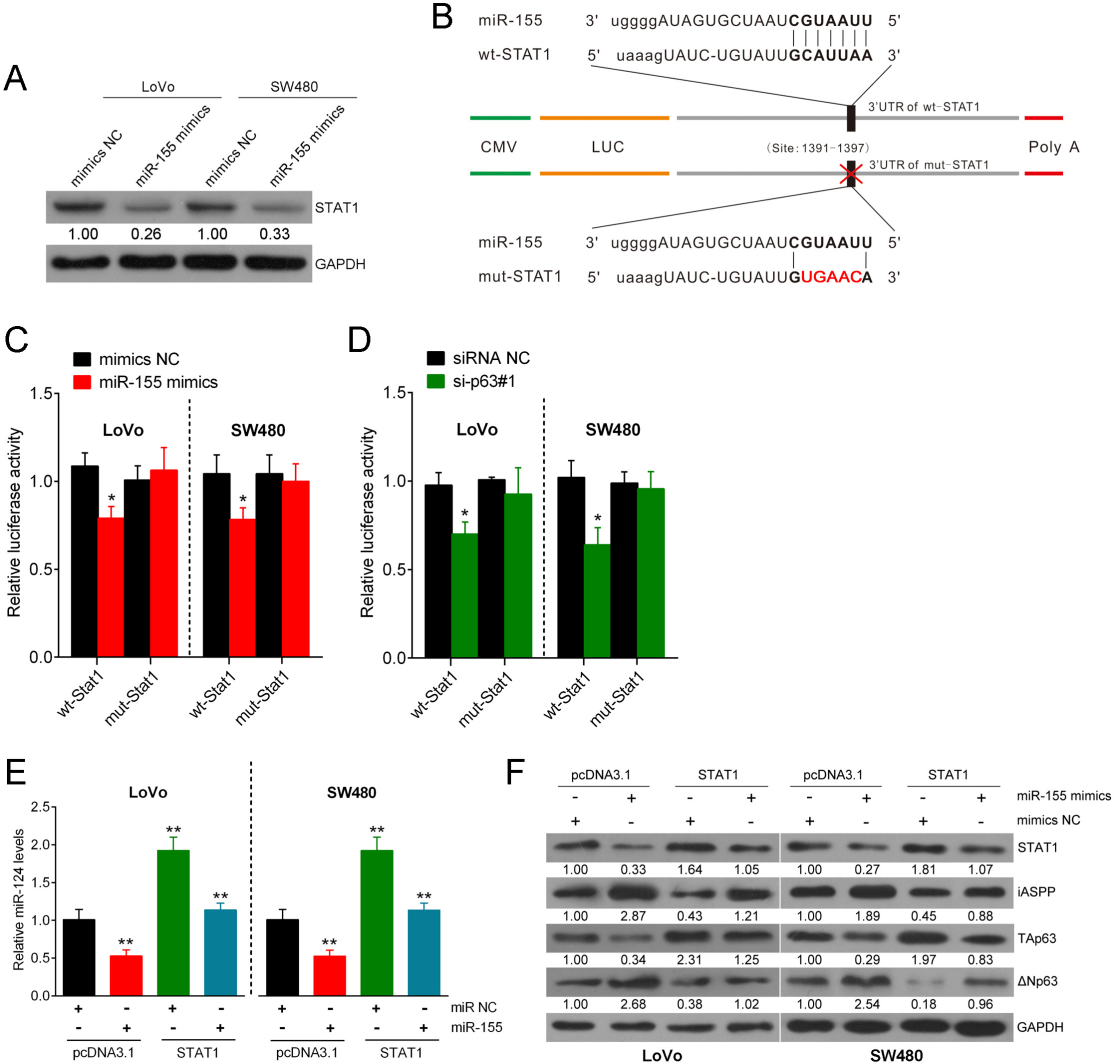
miR-155 targets STAT1 and consequently modulates the miR-124/iASPP/p63 pathway **(A)** LoVo and SW480 cells were transfected with NC mimics or miR-155 mimics, and STAT1 expression was measured. **(B)** A luciferase reporter was used in a construct that incorporated the wild-type or mutant 3′-UTR of STAT1, and in this construct, the sequence corresponding to the seed region was altered. **(C)** The luciferase activity was determined under co-transfection of the wild-type STAT1 luciferase reporter construct or mutant 3′-UTR of STAT1 with either miR-155 mimics or NC mimics into LoVo and SW480 cells. **(D)** The luciferase activity was determined under co-transfection of the wild-type STAT1 luciferase reporter construct or the mutant 3′-UTR of STAT1 with either si-p63#1 or siRNA NC into LoVo and SW480 cells. **(E)** The expression of miR-124 was determined in response to overexpression of STAT1 or miR-155. **(F)** The expression levels of STAT1, iASPP, TAp63, and ΔNp63 were determined using Western blot assay in response to miR-155 overexpression or STAT1 overexpression. The data are presented as the mean ± SD of three independent experiments. *P<0.05, **P<0.01.

According to previous studies, TAp63 regulates CRC cell growth via miR-155. In addition, miR-155 has been reported to interact with TFs including STAT1 [24]. Based on our previous findings that STAT1 induces miR-124 expression and regulates the miR-124/iASPP/p63 axis, we examined whether miR-155 has a functional role in the regulation of the miR-124/iASPP/p63 axis through STAT1. First, we transfected LoVo and SW480 cells with NC mimic or miR-155 mimics and detected STAT1 expression. The expression efficiency was determined using real-time PCR 48 h after transfection (Supplementary Figure 7), and on day 5 and 35 (Supplementary Figure 8) to ensure that miR-155 overexpression was stable. As expected, STAT1 expression was inhibited in CRC cells in response to the overexpression of miR-155 (Figure 4A). To characterize the mechanism whereby miR-155 regulates STAT1, we next explored whether miR-155 directly targets STAT1 via translational repression. We used luciferase reporter constructs incorporating the wild-type or mutant 3′-UTR of STAT1 by altering the sequence corresponding to the seed region (Figure 4B). The co-transfection of the wild-type STAT1 luciferase reporter construct or the mutant 3′-UTR of STAT1 with either miR-155 mimics or the NC mimic in LoVo and SW480 cells markedly attenuated STAT1 3′-UTR luciferase activity. This finding confirms that miR-155 directly suppressed luciferase activity together with the wild-type 3′-UTR of STAT1 but not the mutant version of STAT1 (Figure 4C). Similar results were observed in response to the co-transfection of the wild-type STAT1 luciferase reporter construct or the mutant 3′-UTR of STAT1 with either si-p63#1 or NC siRNA in LoVo and SW480 cells. This result demonstrates that luciferase activity was attenuated together with wild-type STAT1, and wt-STAT1-induced repression of luciferase activity could be abolished by mutant STAT1 (Figure 4D).

Next, we treated cell lines transfected with miR-155 mimics with the STAT1 open reading frame (ORF). STAT1 overexpression restored the inhibitory effect of miR-155 on miR-124 and TAp63 and the inducing effect on iASPP (Figure 4E). In addition, miR-155 overexpression induced ΔNp63 expression, while STAT1 overexpression inhibited ΔNp63 expression (Figure 4F). Taken together, these results show that miR-155 exerts an inhibitory function on the miR-124/iASPP/p63 pathway via the direct targeting of the 3′-UTR of STAT1. Previous studies validated that STAT1 inhibition induces cancer cell proliferation [22, 25], similar to that of miR-155 during the process of cell growth.

### miR-155 regulates CRC growth through the p63 pathway

**Figure 5:**
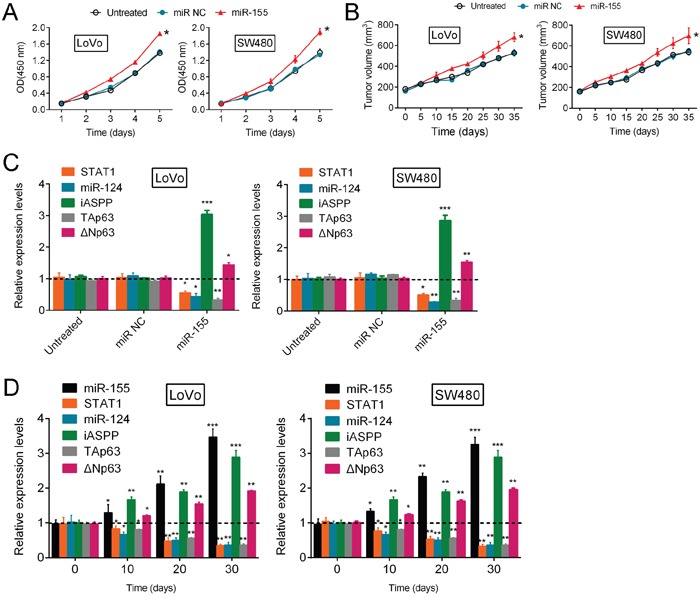
miR-155 regulates CRC growth through the p63 pathway **(A, B)** The proliferation of LoVo and SW480 cells (mean ± SD) and the volume of human tumors (mean ± SEM) were examined in response to miR-155 overexpression. **(C)** The correlation of the expression levels (mean ± SD) of miR-124, STAT1, iASPP, ΔNp63 and TAp63 with the expression level of miR-155 in LoVo and SW480 cells. **(D)** On day 0, 10, 20 and 30 after tumor formation in nude mice, the expression levels (mean ± SD) of miR-155, miR-124, STAT1, iASPP, ΔNp63 and TAp63 were determined by using real-time PCR assays. The data are presented as the mean ± SD of three independent experiments. *P<0.05, **P<0.01, ***P<0.005.

To evaluate the expression levels of STAT1, miR-124, iASPP and p63 in response to the regulation of CRC growth by miR-155, additional studies were performed. The results showed that miR-155 significantly enhanced CRC cell proliferation (Figure 5A) and promoted tumor growth (Figure 5B). Furthermore, miR-155 overexpression significantly reduced the expression of STAT1, miR-124 and TAp63 and enhanced the expression of iASPP and ΔNp63 (Figure 5C). In addition, the expression levels of miR-124, STAT1 and TAp63 were down-regulated in mouse tumors in a time-dependent manner on day 10, 20 and 30 after tumor formation, while the expression levels of miR-155, iASPP and ΔNp63 were up-regulated in mouse tumors in a time-dependent manner on day 10, 20 and 30 after tumor formation (Figure 5D). Taken together, these results suggest that miR-155 promotes CRC cell growth, most likely through the regulation of the STAT1/miR-124/iASPP/p63 pathway.

### STAT1 directly targets miR-124

**Figure 6:**
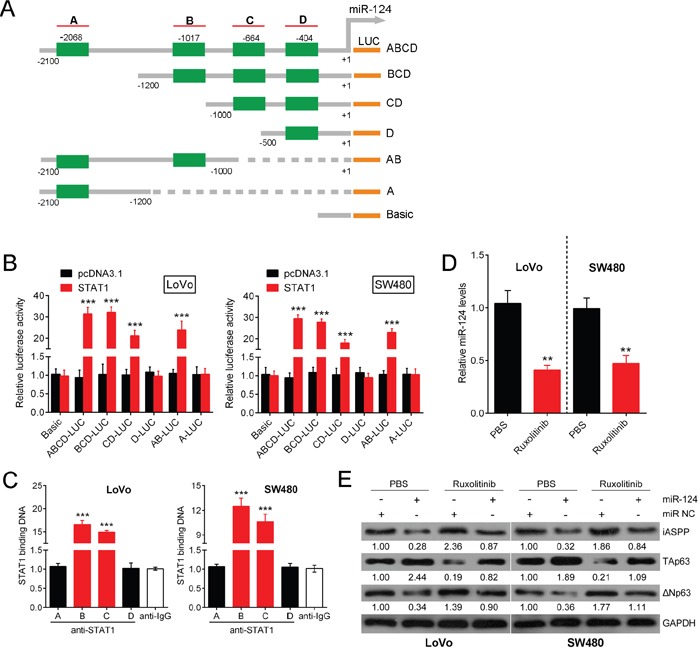
STAT1 directly targets miR-124 Several TFs that might be targets of miR-124 were predicted by TargetScan and JASPAR. **(A)** Four potential binding elements of STAT1 were identified in the promoter region of the miR-124 gene using a bioinformatics analysis. **(B)** Six combinations of these binding elements, each with different lengths (ABCD, BCD, CD, D, AB, A), were sub-cloned into pGL3, and these constructs were co-transfected with Renilla luciferase with or without pcSTAT1, into LoVo and SW480 cells. The luciferase activity was determined respectively. **(C)** A real-time ChIP assay was performed to confirm the binding elements. **(D)** The expression level of miR-124 was determined in response to Ruxolitinib treatment. **(E)** The expression levels of iASPP, TAp63 and ΔNp63 protein were determined using Western blot in response to Ruxolitinib treatment. The data are presented as the mean ± SD of three independent experiments. **P<0.01, ***P<0.005.

As a major oncogene that participates in CRC growth, miR-155 has been reported to regulate the expression of various TFs, including STAT3, FOXO3 and STAT1 [24, 26, 27]. Additionally, TargetScan and JASPAR predicted several TFs, including STAT1, could target miR-124. To examine whether STAT1 binds to miR-124, 4 potential binding elements of STAT1 in the promoter region of the miR-124 gene were identified through a bioinformatics analysis (Figure 6A). Next, we sub-cloned six combinations of these binding elements, each with a different length (ABCD, BCD, CD, D, AB, A), into pGL3 and co-transfected LoVo and SW480 cells with these constructs and Renilla luciferase with or without pcSTAT1. As shown in Figure 6B, the combination of STAT1 with ABCD, BCD, CD and AB significantly increased the luciferase activity compared with pcDNA3.1. When the first binding element A was deleted, luciferase activity was not greatly reduced compared with the full-length promoter. When the first two elements A and B were both deleted, the luciferase activity significantly decreased to levels similar to those observed in the control; however, the luciferase activity remained strong. When the first three elements A, B and C were deleted, the luciferase activity was reduced such that it was not significantly different from that of the control, which suggests that element C might contain a binding site. Moreover, when elements A and B remained, the luciferase activity was strong; when only element A remained, no significant difference in luciferase activity was observed compared with the control, which suggests that element B might contain a binding site. When all STAT1 binding sequences were deleted, a significant reduction in luciferase activity was again detected compared with the full-length promoter (Figure 6B). The results of the ChIP-PCR assay revealed that, compared with the amount of bound IgG, increased STAT1 antibody was bound to elements B and C in the miR-124 promoter (Figure 6C, P<0.005). This result suggests that among the four examined binding elements of STAT1; elements B and C were highly active. These results also indicate that the binding of elements B and C might contribute primarily to STAT-1-induced miR-124 transcription. Moreover, treatment with Ruxolitinib, a known blocker of the JAK/STAT pathway, markedly attenuated the expression of miR-124 and TAp63, while the expression of iASPP and ΔNp63 was enhanced (Figure 6D, 6E). These findings indicate that STAT1 targets miR-124 and induces its expression and that it regulates p63 expression levels most likely through the targeting of miR-124 in CRC cells.

### Roles of the miR-124/p63 feedback loop in CRC growth

**Figure 7:**
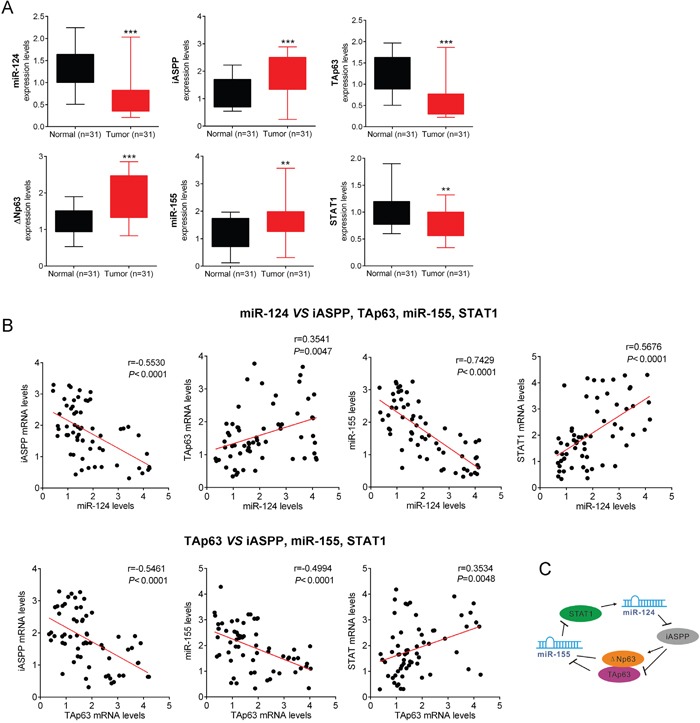
Roles of the miR-124/p63 feedback loop in CRC growth **(A)** The expression levels (mean ± SD) of STAT1, miR-124, iASPP, TAp63/ΔNp63 and miR-155 in CRC samples and adjacent normal tissues were determined. **(B)** The potential correlation among the RNA expression levels of the different members of this circuit was examined in the same samples. **(C)** A schematic representation of the proposed dynamics of a complex molecular self-reinforcing circuit that involves miR-124, iASPP, TAp63/ΔNp63, miR-155 and STAT1 in the regulation of CRC growth. The data are presented as the mean ± SD of three independent experiments. **P<0.01, ***P<0.005.

The miR-124/p63feedback circuit primarily transforms immortalized human colonic cells through the conversion of a transient signal (e.g., acute miR-124 inhibition) into a stable signal. The overexpression of any positive factor (iASPP, ΔNp63, and miR-155) or the inhibition of any negative factor (TAp63, miR-124, STAT1) transforms immortalized colonic cells. To explore the characteristics of the miR-124/p63 feedback, we examined the expression levels of STAT1, miR-124, iASPP, ΔNp63, TAp63 and miR-155 in 31 CRC tissues and adjacent normal tissue samples. The results showed that the expression of STAT1, miR-124 and TAp63 was significantly downregulated, while that of iASPP, ΔNp63 and miR-155 was upregulated in CRC tissues compared with adjacent normal tissues (Figure 7A). Furthermore, in the same samples, we examined the potential correlation among the RNA expression levels of the different members of this circuit. We observed an inverse correlation between miR-124 and iASPP mRNA levels, an inverse correlation between miR-124 and miR-155 levels, a positive correlation between miR-124 and TAp63 mRNA levels, a positive correlation between miR-124 and STAT1 mRNA levels; an inverse correlation between TAp63 mRNA and iASPP mRNA or miR-155 levels, a positive correlation between TAp63 mRNA and STAT1 mRNA levels (Figure 7B). Taken together, these data reveal the dynamics of a complex molecular self-reinforcing circuit that involves miR-124, iASPP, TAp63/ΔNp63, miR-155 and STAT1 in the regulation of CRC growth (Figure 7C).

## DISCUSSION

MiR-124, a major miRNA that is associated with cancer, exerts regulatory effects on various oncogenes and participates in signaling pathways that are closely associated with cancer growth [28–30]. miR-124 exerts tumor suppressive functions on cell proliferation in bladder cancer [31], breast cancer [32] and non-small cell lung cancer [33]. We previously confirmed the effects of the inhibition of iASPP in terms of its regulatory effect of miR-124 on CRC growth [10]. Moreover, the miR-124/iASPP axis has been implicated in stroke [34] and glioblastoma [35]. The oncoprotein inhibitory member of the ASPP family (iASPP) is a key inhibitor of the tumor suppressor p53 in various cancers, including prostate cancer [36], liver cancer [37], lung cancer [38] and glioma [39]. As a crucial member of the p53 family, p63 also plays a key role in tumor development [40]. Recent studies have implicated p63 in a wide range of malignant tumors [41, 42], and among these cancers, the crucial regulatory function of p63 has been reported in CRC [43, 44]. Because the inhibition of TAp63 through iASPP has been identified as an important mechanism by which p63 regulates tumor growth, the iASPP pathway is also a vital regulator of tumor growth [11, 13]. By binding to the apoptosis-regulating proteins such as p53, p63, and p73, among others, ASPP1 and ASPP2 promote apoptosis, while iASPP overexpression typically inhibits apoptotic cell death following DNA damage [45]. Previous studies have demonstrated that transfection of cells with the two isoforms of p63 (TAp63 and ΔNp63) increases the levels of endogenous iASPP expression in HEK293 cells, which suggests a dual regulation between TAp63/ΔNp63 and iASPP [12]. The results of the present study demonstrate the downregulation of iASPP through miR-124 and the upregulation of TAp63; in addition, CRC cell growth was inhibited in response to the overexpression of miR-124. Taken together, these data suggest that miR-124 affects CRC development by targeting iASPP to regulate TAp63/ΔNp63, which is consistent with the mutual regulation that occurs between iASPP and TAp63 in CRC. Thus, we were prompted to identify the potential mechanism whereby the interplay of miR-124, iASPP and p63 regulates CRC growth.

Building on the *in vitro* and *in vivo* findings that the miR-124/iASPP axis regulates CRC cell proliferation via TAp63 and ΔNp63, we assessed the functional roles of TAp63 and ΔNp63 in the regulation of proliferation. Both CRC cell growth and an increase in tumor volume were promoted by p63 inhibition. TAp63 treatment significantly restored the effect of p63 knockdown, but the same effect did not occur with the overexpression of ΔNp63. Similarly, when both isoforms were knocked down, results similar to those after TAp63 knockdown were observed: miR-124 expression caused inhibition and iASPP expression caused induction. Accordingly, ΔNp63 expression altered this alternation and did not affect p63 function.

Recent studies have suggested that miRNAs and TFs may cooperate to modify gene expression in distinct biological and pathological processes. The microRNA-transcription factor feed forward and feedback circuits play important regulatory roles in carcinogenesis [46, 47], and increasing evidence supports the essential functions of these co-regulatory mechanisms in the development of CRC [48, 49]. As a vital transcription factor, p63 is involved in the regulation of various miRNAs, including miR-205 [15], miR-34a [50] and miR-155 [18], which have been identified as oncogenic miRNAs that function in an opposite manner to that of miR-124 in CRC [51]. Li et al. validated that miR-155 regulates the growth and cell cycle progression of colorectal carcinoma cells by targeting E2F2 [52]. Consistent with a previous study that reported that p63 regulates miR-155 expression via two isoforms with diverse functions, TAp63 and ΔNp63 [18], we observed a similar result in the present study and show that TAp63 suppresses miR-155 expression; this finding confirms the regulatory effect of TAp63 on CRC growth via the modulation of miR-155 expression. In addition, a mutual regulatory loop exists between miR-155 and various TFs, including SP1, NFκB1, TP53, MYC, STAT3, FOXO3 and STAT1 [24, 53, 54]. Genome-wide miR profiling has also shown that several miRs, including miR-138, miR-181a, miR-181b, miR-191 and miR-130b, target the 3′UTRs of p63 and regulate p63 expression [55]. Here, we further demonstrate that the iASPP/TAp63 axis regulates miR-155 expression, which prompted us to confirm whether the iASPP/TAp63 axis has a negative feedback regulatory role in the expression of miR-124 through miR-155.

To characterize the potential association between miR-124 and miR-155, a bioinformatics search was conducted (http://alggen.lsi.upc.es/cgi-bin/promo_v3/promo/promo.cgi) to identify TFs that are regulated by miR-124, including Ets-1, Sp1, STAT3 and STAT1. As another important TF involved in carcinogenesis, STAT1 has been reported to interact with miR-155 [24]. In the present study, we confirmed the inhibitory function of miR-155 on STAT1, which leads to miR-155-induced CRC growth. Lin et al. reported a regulatory feedback loop between STAT1 and miR-155-5p that is consistently activated in seven cancer types and that functions to regulate tumor-related biological processes [24]. The consistent results from two independent studies confirmed the mutual regulation of STAT1 and miR-155. Additionally, luciferase and ChIP assays revealed that STAT1 directly targets miR-124. Together, these findings elucidate a feedback loop that consists of miR-124, iASPP, STAT1, miR-155 and p63 and plays an essential role in CRC growth.

To provide a foundation for the determination of whether this miR-124-p63 feedback loop exerts tumor suppressive effects in human colon cancers, we obtained CRC samples and matched adjacent normal tissues to assess the expression of the components of the feedback loop. We observed that miR-124, an important miRNA in solid tumors, was transcriptionally regulated by STAT1 and that miR-124 inhibition downregulated TAp63 expression in CRC cells. Collectively, these results revealed a miR-124-p63 feedback circuit that comprises specific miRNAs and TFs in CRC. The transient inhibition of TAp63 during CRC growth led to increased levels of miR-155 and contributed to the promotion of CRC cell growth. The upregulation of miR-155 in CRC cells attenuated miR-124 expression through a STAT1-dependent mechanism, which facilitated the proliferation of CRC cells. Considering the importance of miR-124 and TAp63 in CRC carcinogenesis, this feedback circuit is not only essential for enhancing our current understanding of colon cancer pathogenesis but also indicates a novel strategy for CRC prevention and therapy.

## MATERIALS AND METHODS

### Reagents

SiRNAs: siRNA negative control (siRNA NC, Ambion Inc., TX, USA) and two different siRNAs against TAp63 and ΔNp63 were synthetized at GeneChem Inc. (Shanghai, China), the oligonucleotides were listed in Supplementary Table 1.

MicroRNAs: miR-124, as-miR-124, miR NC (negative control for miR-124), as-miR NC (negative control for as-miR-124) and miR-155 were purchased from Dharmacon Inc., CO, USA).

cDNA clones: pcDNA3.1-TAp63, pcDNA3.1-ΔNp63, pcDNA3.1-iASPP, pcDNA3.1-STAT1 and pcDNA3.1 vehicle (GeneCopoeia Inc., MD, USA).

The following antibodies were used: CHIP: anti-p63 (CL3748, Sigma-Aldrich, MO, USA); Western blot (WB): iASPP (A4605, Sigma-Aldrich); p63 (sc-367333, Santa Cruz Biotechnology Inc., TX, USA); anti-TAp63 (Cat# AF3482a, Abgent, CA, USA), anti-ΔNp63 (anti-p40; Cat# ABS552, Calbiochem, NJ, USA); STAT1 (S5683, Sigma-Aldrich); GAPDH (ab8245, Abcam, MA, USA).

### Tissue samples

In all, 31 pairs of primary colon cancer and matched adjacent normal tissue samples were collected from patients who underwent surgical resections at the Second Xiangya Hospital of Central South University (Changsha, China). All the human tissues were obtained with informed consent and this study was approved by the Clinical Research Ethics Committee of the Second Xiangya Hospital of Central South University. The samples were snap-frozen in liquid nitrogen and stored at −80°C until further use. This project was approved by the Ethics Committee of the Second Xiangya Hospital of Central South University. The permit number provided by the Ethics Committee is S167.

### Cell culture

The human non-transformed immortalized colonic cell line, HCoEpiC, was purchased from American Type Culture Collection (ATCC) (cat no. CRL-4020), and the human CRC cell lines (LoVo, SW480, SW620, DLD1, HT29 and HCT116) were also purchased from ATCC. The immortalized human colonic epithelial cells HCoEpiC and the human colorectal cancer cell lines LoVo, HT29 and HCT116 were maintained in DMEM (GIBCO, LA, USA) supplemented with 10% FBS, 10 units/ml penicillin and 100 mg/ml streptomycin. SW480, SW620 and DLD1 cells were maintained in RPMI-1640 (GIBCO) supplemented with 10% FBS, 10 units/ml penicillin and 100 mg/ml streptomycin.

### Establishment of stable cell line

The as-miR-124/miR-124 or si-p63#1/si-p63#2 or TAp63/ΔNp63 or miR-155 polyclone stable expression cell lines were established using lentivirus infection and were used for mouse experiments. Briefly, one day before infection, LoVo and SW480 cells were placed onto 6-well plates at 60% confluence. Then, cells were infected with the corresponding lentivirus respectively. After 48h of incubation, cells were passaged three generations with DMEM containing 10% FBS and puromycin (1μg/ml) to obtain the polyclones.

### Mouse experiments

Polyclone as-miR-124/miR-124 or miR-155 or si-p63#1/si-p63#2 or TAp63/ΔNp63 stable expression cell lines were used for the injection of the mice, 10 mice in each group. Stably infected cells (5 × 10^5^ cells per animal) were suspended in 100 μL of serum-free DMEM mixed with 100μL of 10% Matrigel (BD, NJ, USA) and injected subcutaneously into 5-8 week old female BALB/c nude mice. Tumor size was measured on day 0, 5, 10, 15, 20, 25, 30 and 35 after treatment using calipers. Animals were humanely sacrificed after 3-5 weeks depending on tumor volume. The tumor was snap-frozen, and RNA was extracted for measurement of miR-155, miR-124, STAT1, iASPP, ΔNp63, TAp63 and MIR155HG transcript levels. Animal protocols were approved by the Ethics Committee of XiangYa School of Medicine, Central South University.

### RNA extraction and SYBR Green quantitative PCR analysis

Total RNA was extracted from CRC cells using TRIzol reagent (Invitrogen, CA, USA). Total RNA was extracted from tissue samples following the protocol by Helen Pearson [23]. The expression levels of miR-124 and miR-155 were detected using the Hairpin-it™ miRNAs qPCR Kit (Genepharma, Shanghai, China). The expression of RNU6B served as an endogenous control. IASPP, p63 and STAT1 expression was measured using a SYBR Green qPCR assay (Takara, Dalian, China). The data were processed according to the 2^−ΔΔCT^ method. The primers were shown in Supplementary Table 1.

### CCK-8 cell proliferation assay

Cell proliferation rates were measured using the Cell Counting Kit-8 (CCK-8) (Beyotime, Hangzhou, China) methods. Approximately 0.5 × 10^4^ cells were seeded in each 96-well plate for 24 h, after which, they were transfected with the indicated miRNA or siRNA, and further incubated for 24, 48, 72 or 96 h. CCK-8 reagent (10 μL) was added to each well 1 h before the endpoint of each incubation period. The OD_450_ value was determined for each well using a microplate reader.

### Western blot analysis

The cells were lysed in 150 mmol/L NaCl, 1% Triton-X, 50 mmol/L Tris-HCl (pH 8) with complete protease inhibitor cocktail (Roche, Basel, Switzerland). The samples were then sonicated and centrifuged. Clarified cell lysates or immunoprecipitated protein samples were resolved by SDS-PAGE and transferred onto Hybond-C Extra membranes (Amersham Biosciences, IL, USA). Nonspecific binding was blocked with 5% milk in TBST buffer for 2 h. The blots were probed with 1:1000 diluted iASPP (mouse monoclonal), p63 (mouse monoclonal), anti-TAp63 (rabbit polyclonal), anti-ΔNp63 (rabbit polyclonal), STAT1 (rabbit polyclonal) and GAPDH (mouse monoclonal) at 4°C overnight, then incubated with 1:5000 horseradish peroxidase conjugated secondary antibody for 2 h. An enhanced chemiluminescence (ECL) detection system (Amersham Biosciences) was used to visualize signals following standard protocols. GAPDH served as an endogenous protein control for normalization.

### Luciferase reporter assay

LoVo and SW480 cells were transfected with miR-155 mimics or si-p63 and pGL3-Basic luciferase reporter vector harboring the miR-155 target sequence of STAT1 3′UTR. After 24 h, the activities of firefly luciferase and Renilla luciferase were measured in the cell lysates using a Dual-Luciferase Assay System (Promega, WI, USA). For the luciferase transcription reporter assay, miR-124 gene promoter sequences (WT or site deletion) were cloned into the promoter region of the pGL3-Basic luciferase reporter vector. LoVo and SW480 cells were co-transfected with the luciferase reporter vector and pcDNA 3.1-STAT1 expression vector. The luciferase activity was measured as described above. The primers for plasmid construction were showed in Supplementary Table 1.

### Chromatin immunoprecipitation (ChIP)

Briefly, the treated cells were cross-linked with 1% formaldehyde, sheared to an average size of 400 bp DNA, and immunoprecipitated using antibodies against p63 (anti-p63, clone 4YA3, Sigma-Aldrich). The ChIP-PCR primers were designed to amplify the promoter regions containing putative p63 binding sites within miR-155, as previously described (MIR155-p63BS-F and MIR155-p63BS-R are used in the ChIP assay, showed in Supplementary Table 1). A positive control antibody (RNA polymerase II) and a negative control non-immune IgG were used to demonstrate the efficacy of the kit reagents (Epigentek Group Inc., NY, USA, P-2025-48). The immunoprecipitated DNA was subsequently cleaned, released, and eluted. The eluted DNA was used for downstream applications, such as ChIP-PCR. The fold-enrichment (FE) was calculated as the ratio of the amplification efficiency of the ChIP sample to that of the non-immune IgG. The amplification efficiency of RNA Polymerase II was used as a positive control. FE% = 2 (IgG CT-Sample CT) × 100%.

### Statistical analyses

Data from 3 independent experiments were expressed as the mean ± SD and processed using SPSS17.0 statistical software. The expression levels of miR-124, iASPP mRNA, TAp63 mRNA and ΔNp63 mRNA in CRC tissues and matched adjacent normal tissues were compared using Wilcoxon's paired test. Differences among the groups in the above assays were estimated using Student's t-test or one-way ANOVA. A *P* value <0.05 was considered significant.

## SUPPLEMENTARY MATERIALS FIGURES AND TABLES




