# Supplementary material for: The miR-124-p63 feedback loop modulates colorectal cancer growth
Source: Oncotarget. 2017 Mar 16;8(17):29101–15. doi: 10.18632/oncotarget.16248 (PMC5438716; doi:10.18632/oncotarget.16248)
Supplement: Supplementary file 1 [file oncotarget-08-29101-s001.pdf]

The miR-124-p63 feedback loop modulates colorectal cancer growth

SUPPLEMENTARY MATERIALS

SUPPLEMENTARY FIGURES AND TABLE

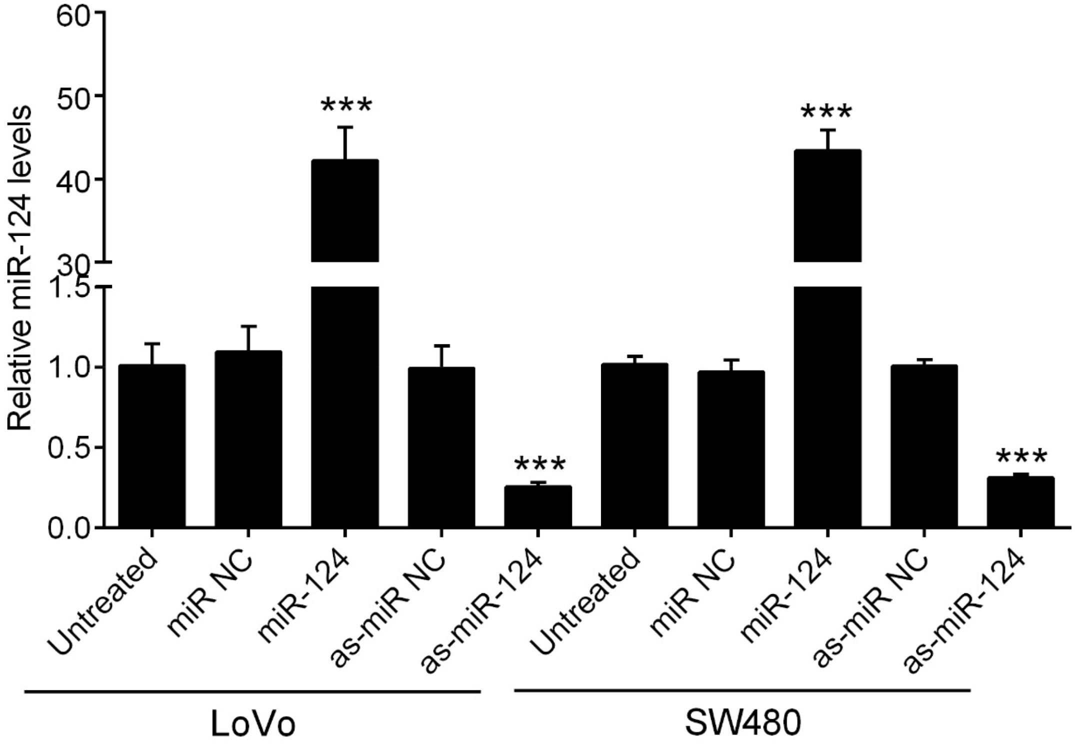

Supplementary Figure 1: Relative miR-124 expression in LoVo and SW480 cell lines as measured by real-time PCR.

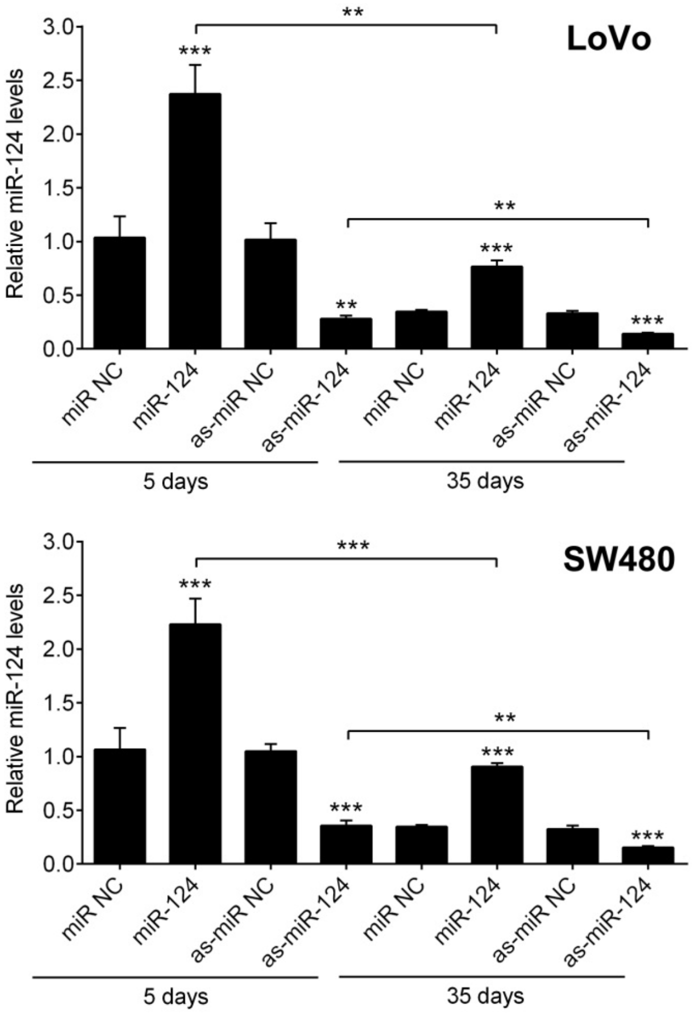

Supplementary Figure 2: p63 isoform expression in LoVo and SW480 cell lines as validated by Western blot.

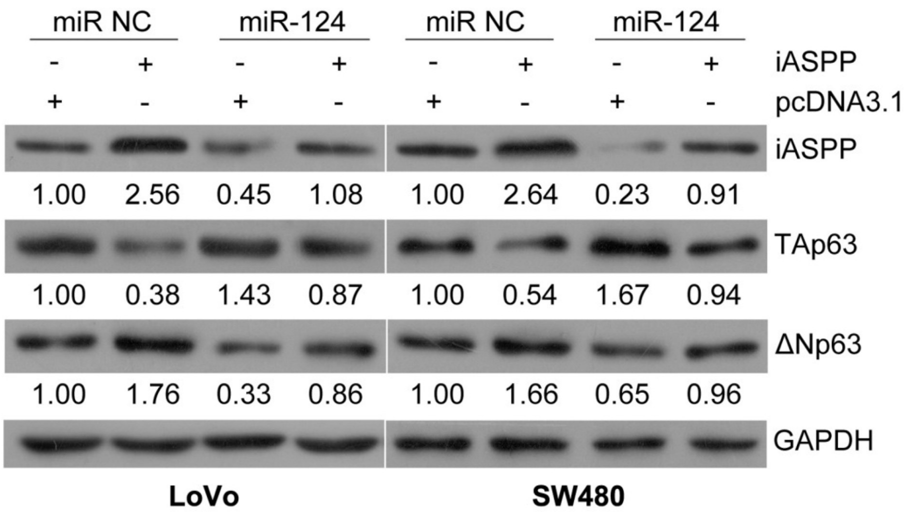

Supplementary Figure 3: Relative miR-155 expression in LoVo and SW480 cell lines as measured by real-time PCR.

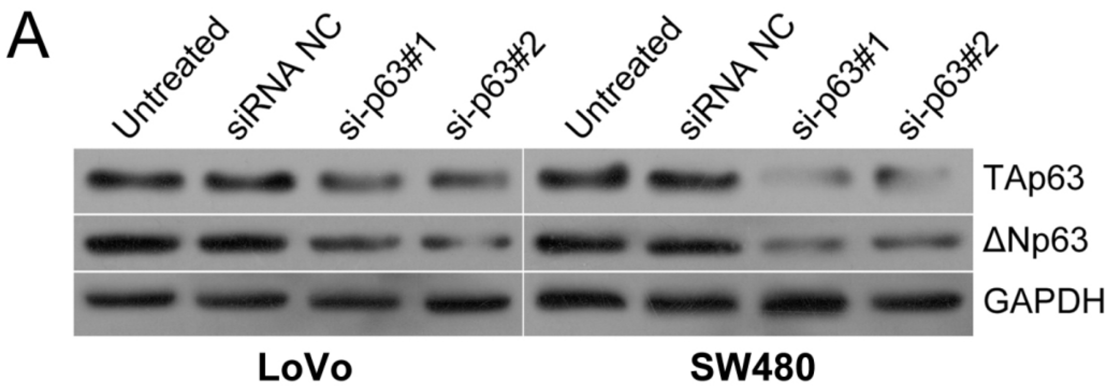

Supplementary Figure 4: The inhibitory efficiency of si-p63#1 and si-p63-#2 on the protein levels of TAp63 and ΔNp63 as verified by using Western blot assays.

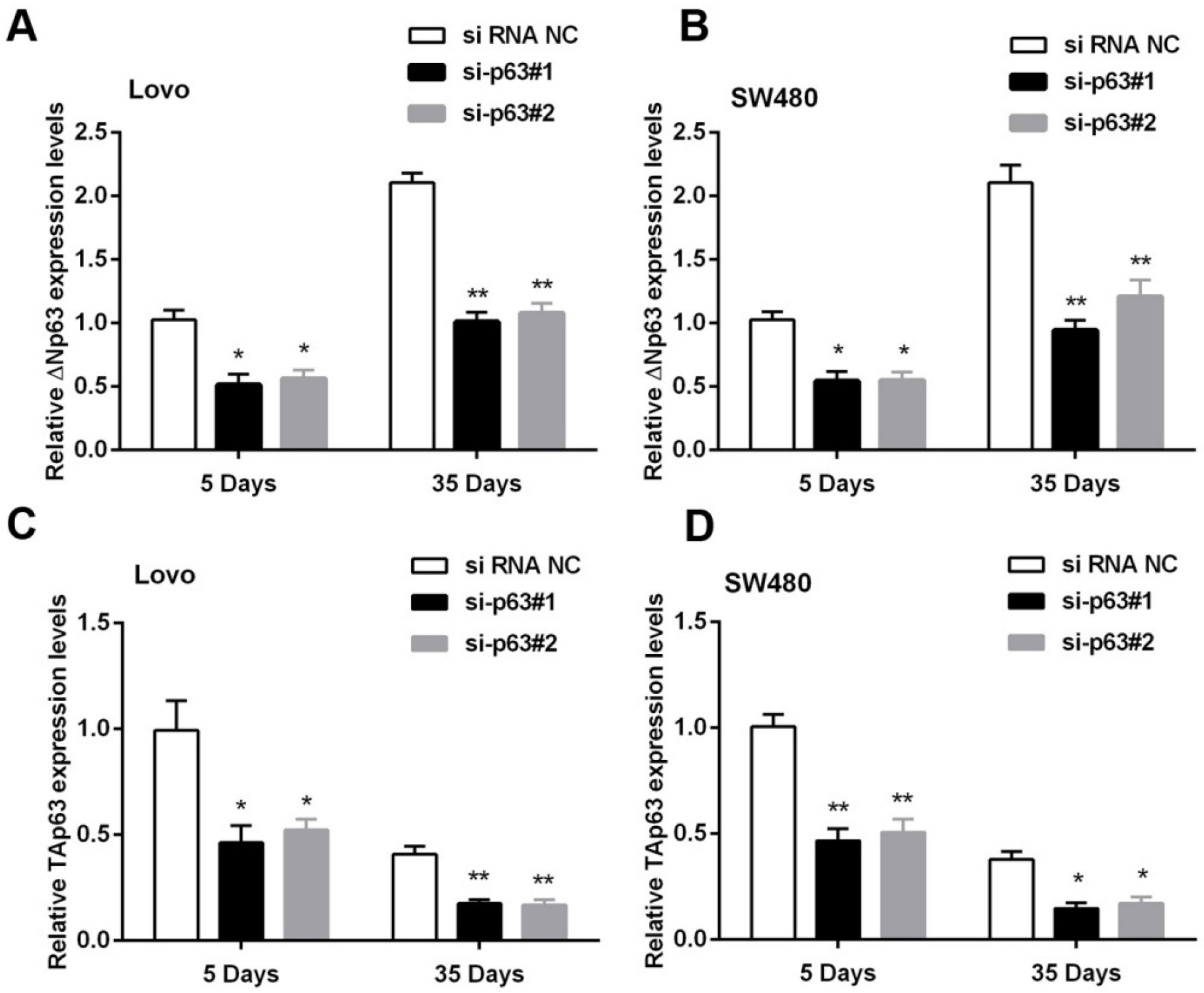

Supplementary Figure 5: On day 5 and day 35 after tumor formation in nude mice, the expression levels of p63 were verified by RT-PCR.

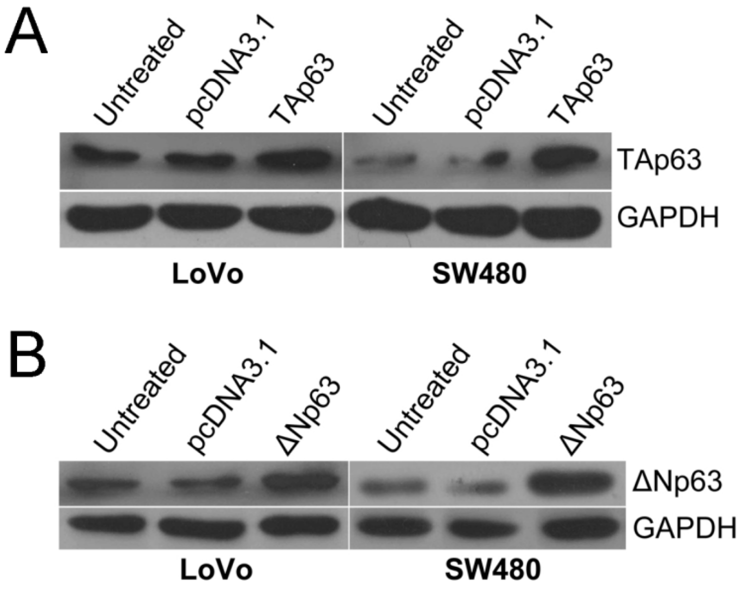

Supplementary Figure 6: The forced TAp63 and ΔNp63 expression was achieved by TAp63 and ΔNp63 vectors, respectively, as verified by using Western blot assays.

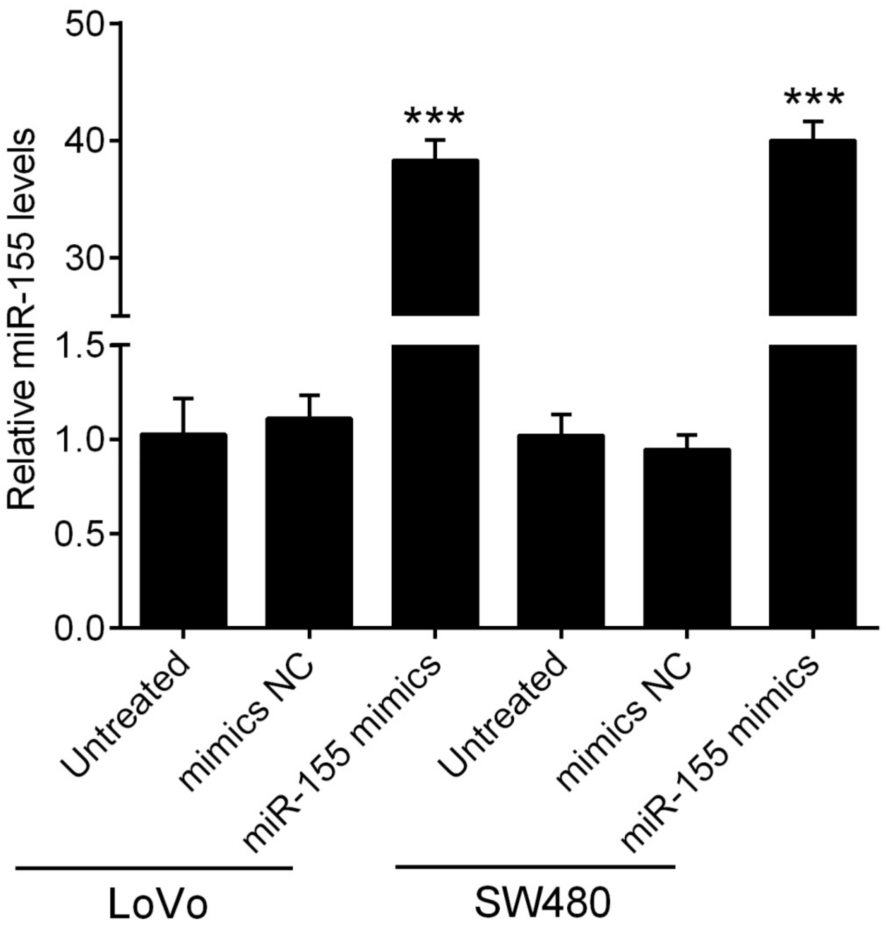

Supplementary Figure 7: MiR-155 mimics was transfected into LoVo and SW480 cells to achieve miR-155 overexpression. The transfection efficiency was verified by using real-time PCR.

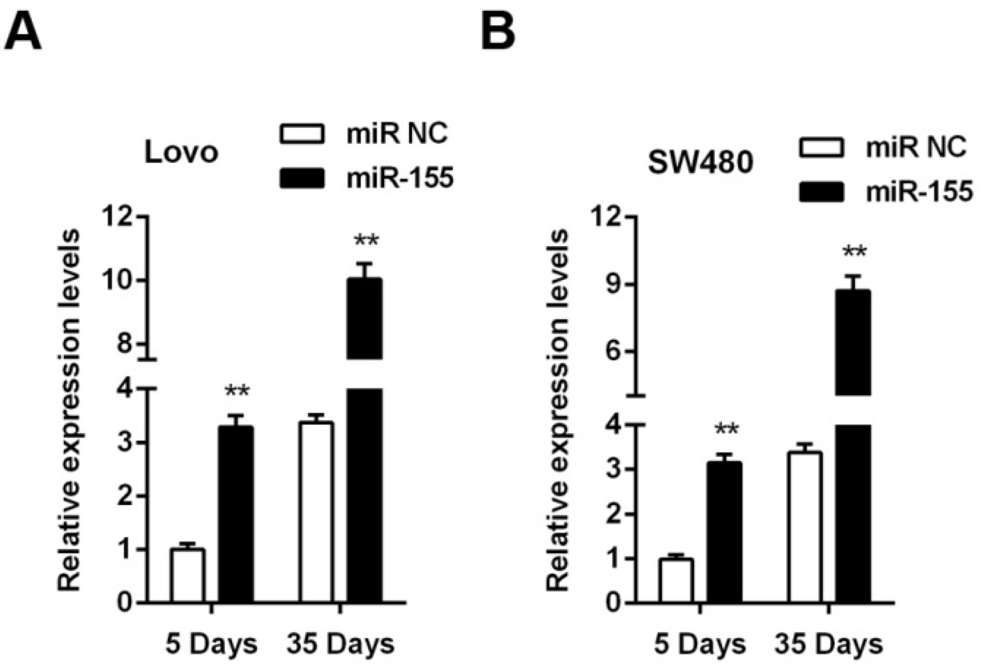

Supplementary Figure 8: The expression levels of miR-155 on day 5 and 35 after transfection were determined to ensure that miR-155 overexpression was stable.

Supplementary Table 1: Oligonucleotides

See Supplementary File 1
