# Supplementary material for: The miR-124-p63 feedback loop modulates colorectal cancer growth
Source: Oncotarget. 2017 Mar 16;8(17):29101–15. doi: 10.18632/oncotarget.16248 (PMC5438716; doi:10.18632/oncotarget.16248)
Supplement: Supplementary file 2 [file oncotarget-08-29101-s002.docx]

Supplementary table 1 - Oligonucleotides

| Primer | Sequence |
| --- | --- |
| iASPP realtime forward | GGCGGTGAAGGAGATGAAC |
| iASPP realtime reverse | TGATGAGGAAATCCACGATAGAG |
| TAp63 realtime forward | GGACTGTATCCGCATGCAG |
| TAp63 realtime reverse | GAGCTGGGCTGTGCGTAG |
| DNp63 realtime forward | TTGTACCTGGAAAACAATGCCC |
| DNp63 realtime reverse | GGGACTGGTGGACGAGGAG |
| STAT1 realtime forward | TCAGACCACAGACAACCT |
| STAT1 realtime reverse | GTCGCCAGAGAAGATGAA |
| GAPDH realtime forward | CTCAGACGGCAGGTCAGGTCCACC |
| GAPDH realtime reverse | CCACCCATGGCAAATTCCATGGCA |
| MIR155HG-F | GCGCCTTAATGCTAATCGTGAT |
| MIR155HG-R | GTGCAGGGTCCGAGGT |
| MIR155-p63BS-F(for ChIP) | TCATGTCATTCTTAATTGCAGGTTTTGGC |
| MIR155-p63BS-R(for ChIP) | ACCTGGGGGAAAGTACCAGTTTCT |
| si-p63#1 | cagaagaatggtacaaatccaag |
| Si-p63#2 | cccttaaaggaaccaatgagtcc |
| Wt-STAT1-3’UTR-F | AAACTCGAGattaagagatgggtttga |
| Wt-STAT1-3’UTR-R | AAAGCGGCCGCgcaattagaaacaatatt |
| Mut-STAT1-3’UTR-F | taaagtatctgtattgcattaaatataatatgcacacagt |
| Mut-STAT1-3’UTR-R | actgtgtgcatattatatttaatgcaatacagatacttta |
| MIR-124 promoterABCD-F | AAAGCTAGCcagttcgggcttggccgt |
| MIR-124 promoterABCD-R | AAAAGATCTcagccccattcttggcat |
| MIR-124 promoterBCD-F | AAAGCTAGCtacacgagtgacctttta |
| MIR-124 promoterBCD-R | AAAAGATCTcagccccattcttggcat |
| MIR-124 promoterCD-F | AAAGCTAGCgagctatctaggtcatta |
| MIR-124 promoterCD-R | AAAAGATCTcagccccattcttggcat |
| MIR-124 promoterD-F | AAAGCTAGCggggaggcgaggcgcgggga |
| MIR-124 promoterD-R | AAAAGATCTcagccccattcttggcat |
| MIR-124 promoterAB-F | AAAGCTAGCcagttcgggcttggccgt |
| MIR-124 promoterAB-R | AAAAGATCTtaatgacctagatagctc |
| MIR-124 promoterA-F | AAAGCTAGCcagttcgggcttggccgt |
| MIR-124 promoterA-R | AAAAGATCTtaaaaggtcactcgtgta |
| Overexpression-STAT1-F | AAAGGATCCatgtctcagtggtacgaa |
| Overexpression-STAT1-R | AAAGCGGCCGCctatactgtgttcatcat |
| MIR124 promoterA-F(for ChIP) | cagttcgggcttggccg |
| MIR124 promoterA-R(for ChIP) | gaaatgaaaaggatcgaga |
| MIR124 promoterB-F(for ChIP) | ctctcctccctccctccccc |
| MIR124 promoterB-R(for ChIP) | tgacctagatagctcatg |
| MIR124 promoterC-F(for ChIP) | ccccactcagcgatggag |
| MIR124 promoterC-R(for ChIP) | aagaagagagcgaaggac |
| MIR124 promoterD-F(for ChIP) | attaacacgggggaggca |
| MIR124 promoterD-R(for ChIP) | ccatcctttcgcatccag |
